# The Future of Diabetes Prevention: A Call for Papers

**DOI:** 10.1371/journal.pmed.1001966

**Published:** 2016-02-16

**Authors:** The PLOS Medicine Editors

## Abstract

*PLOS Medicine* editors and guest editors announce a call for papers for submissions for a special issue on diabetes prevention.

*PLOS Medicine*, now in our 12th year, publishes research across the spectrum of translation, from lab to clinic to epidemiology and public health. As editors of a journal with such a potentially broad scope but a limited volume of monthly articles, we are continually challenged to decide which topics to emphasize. In 2016, we have decided to take a new approach by publishing occasional special monthly issues on topics that we believe merit particular attention in the most accessible of the world’s major medical journals. The first of these, planned for the summer of 2016, will be on diabetes prevention.

We are thrilled to be working with two guest editors for the special issue, Professor Nick Wareham (MRC Epidemiology, University of Cambridge, United Kingdom) and Professor William Herman (Internal Medicine and Epidemiology, University of Michigan, United States). Both of our guest editors have exceptional track records in diabetes research, and their input will be invaluable as we put together the special issue that will consist of exceptional research as well as commissioned essays and editorials.

Why diabetes prevention? Diabetes is now firmly recognized as a global problem, with recent estimates of a staggering 410 million people with diabetes mellitus in 2013 [1]. Furthermore, projections from the International Diabetes Foundation suggest that by 2040 the global burden of diabetes may be as high as 642 million, which would mean that one in ten adults will be living with diabetes [2]. The high risk of disabling and life-threatening complications resulting from poor control of blood glucose and the enormous individual and societal costs associated with these complications demonstrate the urgent need for more effective approaches to prevention.

While providing care for those with diabetes is an important facet of the response to the diabetes epidemic, as Professor Wareham recently noted, “our immediate public health goal should be to limit and ideally reverse the increase in the incidence of the condition, rather than to impact on the overall prevalence since this is likely to continue to rise in the future” [3]. Furthermore, he highlights that “there is no single intervention that can effect such population-level changes; instead, a wide range of different approaches are needed” [3].

We should also be under no illusion that diabetes represents a disease that primarily affects those in high-income countries. According to Professor Herman, “approximately 75% of people with diabetes live in low- and middle-income countries, and the largest increases in diabetes prevalence are predicted to occur in regions where economies are moving from low- to middle-income levels.”

*PLOS Medicine* has a strong interest in noncommunicable diseases and their societal drivers. Of course, we also believe that a key to ensuring important research can be translated into action is to reduce the barriers to dissemination and use by publishing in an open access venue. Public access is particularly important for research on societal problems of the scope and scale of the diabetes epidemic, which require the engagement of partners beyond the scientific research community. Consistent with *PLOS Medicine’s* usual policy, the special issue will not receive sponsorship or advertising from companies that produce drugs or medical devices.

For researchers interested in having their work considered for the Diabetes Prevention Special Issue, the deadline for submissions is **Friday, 4 March 2016**. Authors of submissions that represent sound science but are not selected for the issue itself may be offered transfer of their manuscript to *PLOS ONE*, with inclusion in an overall Collection on Diabetes Prevention that will include papers published in both journals.

For the special issue we will consider laboratory translational or clinical studies describing policy or practice insights into diabetes prevention, including randomized trials, significant observational studies, systematic reviews and meta-analyses, diagnostic/prognostic evaluations, modelling studies, cost-effectiveness analyses, and natural experiments. In particular, we would welcome submissions from the following areas:

- Individual-level interventions aimed at prevention of type 2 diabetes
- Societal approaches to the prevention of type 2 diabetes
- Early detection of type 2 diabetes
- Prevention in resource-limited settings
- Studies of the genetic basis of obesity and type 2 diabetes that have direct implications for prevention
- Type 1 and gestational diabetes prevention

Please submit your manuscript through our submissions site at the following address: http://journals.plos.org/plosmedicine/s/submit-now

Authors are not required to send a presubmission inquiry when submitting a manuscript for the Diabetes Prevention Special Issue. Please indicate in your cover letter that you would like the full manuscript to be considered for the special issue. If you would like to inquire about the suitability of a manuscript for consideration, please email plosmedicine@plos.org.
